# A 52 month follow-up of functional decline in nursing home residents – degree of dementia contributes

**DOI:** 10.1186/1471-2318-14-45

**Published:** 2014-04-10

**Authors:** Anne-Sofie Helvik, Knut Engedal, Jūratė Šaltytė Benth, Geir Selbæk

**Affiliations:** 1 Department of Public Health and General Practice, Faculty of Medicine, Norwegian University of Science and Technology (NTNU), Trondheim, Norway; 2 St Olav’s University Hospital, Trondheim, Norway; 3 The Norwegian Centre for Dementia Research, Vestfold Health Trust, Tønsberg, Norway; 4 Institute of Clinical Medicine, Campus Ahus, University of Oslo, Oslo, Norway; 5 HØKH, Research Centre, Akershus University Hospital, Lørenskog, Norway; 6 Centre for Old Age Psychiatric Research, Innlandet Hospital Trust, Ottestad, Norway; 7 Akershus University Hospital, Lørenskog, Norway

## Abstract

**Background:**

Few have studied how personal activities of daily living (P-ADL) develop over time in nursing home residents with dementia. Thus, the aim was to study variables associated with the development of P-ADL functioning over a 52-month follow-up period, with a particular focus on the importance of the degree of dementia.

**Method:**

In all, 932 nursing home residents with dementia (Clinical Dementia Rating–CDR- Scale ≥1) were included in a longitudinal study with four assessments of P-ADL functioning during 52 months. P-ADL was measured using the Lawton and Brody’s Physical Self-Maintenance Scale. Degree of dementia (CDR), neuropsychiatric symptoms and use of psychotropic medication were assessed at the same four time points. Demographic information and information about physical health was included at baseline. Linear regression models for longitudinal data were estimated.

**Results:**

Follow-up time was positively associated with a decline in P-ADL functioning. Degree of dementia at baseline was associated with a decline in P-ADL functioning over time. The association between degree of dementia and P-ADL functioning was strongest at baseline, and then flattened over time. A higher level of neuropsychiatric symptoms such as agitation and apathy and no use of anxiolytics and antidementia medication were associated with a decline in P-ADL functioning at four time points. Higher physical co-morbidity at baseline was associated with a decline in P-ADL functioning.

**Conclusion:**

P-ADL functioning in nursing home patients with dementia worsened over time. The worsening was associated with more severe dementia, higher physical comorbidity, agitation, apathy and no use of anxiolytics and antidementia medication. Clinicians should pay attention to these variables (associates) in order to help the nursing home residents with dementia to maintain their level of functioning for as long as possible.

## Background

Dementia is, in most cases, characterized by a progressive decline in cognitive function. In Europe, it is estimated that between six to ten million persons have dementia [1]. The prevalence of dementia increases with age, from approximately 1.5% in persons aged 60–69 years to 40% in persons 90 years and older [2]. The prevalence of dementia is found to be high in nursing home residents in the Western countries [3-6]. Studies in Norway have indicated that more than 80% of nursing home residents have dementia [7,8].

In general, dementia will negatively affect the person’s ability both to function adequately in everyday life and to perform personal activities of daily living (P-ADL), such as bathing, dressing, eating, grooming, ambulation/transferring and toileting [9-17].

Dementia and the accompanying P-ADL impairment increase the risk of being admitted to nursing home care [18,19] as increasing P-ADL impairment increases the need of care. Lower P-ADL functioning increases the burden for the patients, their family, the professional caregivers and society as a whole [15,20,21]. Furthermore, P-ADL impairment in older persons increases the risk of mortality [22-25].

Up to now, relatively few studies have explored how P-ADL functioning changes over time in nursing home residents with dementia [26]. An early study explored P-ADL decline in nursing home residents without assessing dementia and or cognitive impairment [22]. Others have explored if the presence of dementia (yes/no) in nursing home residents at baseline was important for later P-ADL decline [23,27,28]. Diverging results were reported. Furthermore, prospective register studies of nursing home residents with and without dementia have studied the association between P-ADL decline and the degree of cognitive impairment with a follow-up period of three months to one year. These studies found that a decline of P-ADL functioning was explained by the degree of P-ADL impairment and degree of cognitive impairment at baseline [29-32]. None of the register studies explored the importance of medical co-morbidity to a P-ADL decline.

A recent six-month follow-up study of long-term residents with dementia reported that a decline in P-ADL functioning was associated with the patients’ degree of dementia [26]. However, this study had excluded the residents with physical co-morbidities and did not study the influence of neuropsychiatric symptoms on P-ADL development. Information about medical co-morbidity and neuropsychiatric symptoms should be included, since studies report that such symptoms may have importance for P-ADL functioning [31,33,34]. Other risk factors for a decline in P-ADL functioning over time in nursing home residents may be age, ethnicity, gender, marital status and education [22,27,28,31,32,35]. Protective factors for a decline in P-ADL have also been reported, such as the long-term use of antidementia medication [20,36,37].

A number of disease specific and generic P-ADL indexes have been developed, validated and found sensitive to small but significant changes in persons’ ability to perform P-ADL [20,38]. Even so, several studies of P-ADL decline in nursing home residents have used single items [26,32,33,35] rather than P-ADL indexes covering a range of P-ADL functions [23,31]. The Physical Self-Maintenance Scale is one of the shorter recommended P-ADL indexes [20], which has been frequently used in Scandinavian studies [7,8,39,40].

To our knowledge no long-term follow-up of nursing home residents has been conducted including both a measure of cognition and physical co-morbidities. Thus, our aim was to study the association between degree of dementia, both at baseline and over more than four years, and the development in P-ADL functioning measured by the Physical Self-Maintenance Scale (P-ADL score), adjusting for a number of other variables know to have an influence on P-ADL functioning in nursing home residents with dementia. We hypothesize that P-ADL functioning in persons with dementia living in nursing homes will decline over time, and that this decline will be associated with a worsening of dementia, the neuropsychiatric symptom load, medication and physical co-morbidity.

## Method

### Design

This was a 52 months prospective study with four assessments. Baseline assessment took place between November 2004 and January 2005. The follow-up assessments took place after 12, 31 and 52 months.

### Participants

All residents in 26 nursing homes in 18 municipalities and four counties in Norway with a stay of a minimum of 14 days were asked to participate in the study. The municipalities were chosen to make the sample representative in terms of municipality size [8]. In all, 1165 residents were eligible. Two residents or their next of kin refused participation, four had important missing information, and 233 had no dementia according to the Clinical Dementia Rating (CDR) scale, i.e. CDR < 1 [41]. Thus, 932 residents were included in the longitudinal study of P-ADL.

### Measures

The Demographic information age, level of education, marital status and length of stay at inclusion, was collected from the medical records.

The Medical health information was collected from the medical records with the use of diagnoses in the International Statistical Classification of Diseases and Related Health problems 10^th^ Revision (ICD-10). The diagnoses were sorted into diagnostic groups, i.e. stroke-, cardiovascular-, pulmonary-, musculoskeletal-, digestive-, endocrine-, neurological-, genito-urinal- and neoplasm diagnoses [42]. A co-morbidity index was calculated by summing up the occurrence of diagnoses (yes/no) in each of the nine groups of diagnoses.

Sensory loss was registered by two single items for vision and hearing impairment, respectively, i.e. perceived severely impaired or not. No specific measurement was used and the occurrence of vision and hearing impairment are based on the primary professional caregiver’s clinical judgement.

Dementia was assessed using the Clinical Dementia Rating (CDR) scale which covers six domains (memory, orientation, judgment and problem solving, community affairs, home and hobbies, and personal care) with five response categories (0, 0.5, 1, 2, 3) [41,43]. The total score is calculated by means of an algorithm giving priority to memory [41]. Residents with a total score of one or higher were regarded as having dementia [44-46]. In the analysis we used the CDR sum score (sum of boxes), which ranges from 0 to 18 with a higher score indicating greater dementia severity. The correlation between the CDR score and the CDR sum of boxes is high. However, due to an increased range of values, the CDR sum of boxes offers important advantages when analyzing the data. [47].

Neuropsychiatric symptoms were assessed using the Neuropsychiatric Inventory (12-item NPI) [48] in a translated and validated Norwegian version [49]. The 12-item inventory covers the following symptoms: delusion, hallucination, agitation/aggression, disinhibition, irritability/lability, depression/dysphoria, anxiety, apathy/indifference, aberrant motor behaviour, sleep and night-time behaviour disorder and appetite/eating changes. For each symptom, severity (score 1–3) multiplied by frequency (score 1–4) gave a score ranging from one to twelve. Based on a previous principal component analysis, psychosis (delusions, hallucination), agitation (agitation/aggression, disinhibition, irritability) and affective (depression, anxiety) sub-syndrome scores were formed by summing the score of the included items [50-53].

Psychotropic medications were grouped according to the ATC code, into antipsychotics (N05A except lithium), antidepressants (N06A), anxiolytics (N05B), hypnotics/sedatives (N05C) and antidementia medication (N06D) (yes versus no). The information was collected from the medical record of each resident.

Level of functioning in personal Activities of Daily Living (P-ADL), the dependent variable, was measured by the Physical Self-Maintenance Scale (six items, score range 6–30, i.e. P-ADL scale score) [54]. High scores indicate a lower level of functioning.

### Procedure

Prior to the study, 16 research assistants (all registered nurses) attended a two-day training program on how to conduct the interview. A one-day training program was carried out prior to each follow-up assessment. The project leader (GS) was available for consultation while the data were collected. The research assistants collected the data by means of a standardized interview with the primary professional caregiver and by extracting information from the medical records.

Study information was given to the patient and their family members. An explicit consent was not required for enrollment, but the patients and their next of kin were informed in writing that they could refuse to participate at any stage of the study. The study, and the procedure for information and the right to decline participation were approved by the Regional Ethics Committee of Eastern Norway in 2004, the Data Inspectorate and the Directorate for Health and Social Affairs.

### Data analysis

Continuous socio-demographic and clinical characteristics were presented as means and standard deviations (SD), while frequencies and proportions were used for categorical characteristics. A linear regression model was estimated to assess the development in P-ADL throughout the follow-up period of 52 months. As P-ADL consists of repeated measurements, the assumption of independent observations required for ordinary linear regression was violated. Therefore, a linear mixed model containing both linear and second–order time components as fixed effects (growth model) as well as random effects for intercepts and slopes for time was estimated first. Such a model accounts for correlations due to repeated measurements and accommodates any degree of imbalance in longitudinal data. That is, the model is particularly suited for analysing data with missing values and drop-outs due to death, for instance. The SAS MIXED procedure was used to fit the model. The main independent variable, degree of dementia assessed with CDR sum of boxes, and interaction term between degree of dementia and time were then entered into the model as fixed effects. Two different models with respect to adjustment were further estimated. In both models, adjustment variables were demographic characteristics (length of stay prior to baseline, age, gender, education, and marital status), physical health information (co-morbidity index, severe sensory loss), neuropsychiatric symptoms and use of psychotropic medications. Length of stay, age, and gender were defined to be the confounders, while the other adjustment variables were defined as secondary independent variables. The first model contained adjustment variables all measured at baseline. The second model included demographic characteristics, somatic health difficulties at baseline, while neuropsychiatric symptoms of dementia and use of psychotropic medications were entered as longitudinal variables. The following modelling strategy was employed. Firstly, a model containing all defined secondary independent variables was reduced by applying Akaike’s Information Criteria (AIC). The least significant secondary independent variables were removed from the model one at a time and AIC was calculated at each step. The model with the smallest value of AIC was chosen. Secondly, the CDR sum of boxes, interaction term between the CDR sum of boxes and time, and the three confounders were entered into the reduced model and a final model was estimated. Lastly, to assess eventual gender-specific differences, interactions between gender and CDR sum of boxes, age, and education were entered into the final model. However, none of them were significant and therefore excluded. The results of the regression analysis were tabulated as coefficients with the corresponding 95% confidence intervals (CI). Regression coefficients were used to estimate the P-ADL score for each time point from an unadjusted model as well as from both adjusted models at the median baseline CDR sum of boxes value and were presented graphically.

Analyses were performed in SAS v9.2 and SPSS v20. P-values below 0.05 were considered statistically significant. All tests were two-sided.

## Results

### Sample characteristics

**Figure 1:**
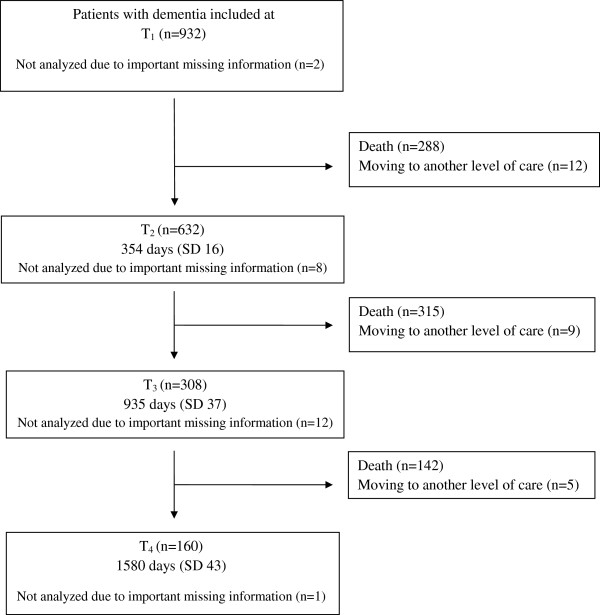
**Flow chart of participants from baseline (T**_**1**_**) to last follow-up up (T**_**4**_**), with mean (SD) follow-up time at each assessment.**

At baseline, the mean (SD) age and length of stay for the participants were 84.5 (7.5) years and 932 (910.1) days, respectively (see Table 1). In all, 246 (26.4%) of the residents were men and the mean score of the co-morbidity index was 1.8 (SD 1.3, ranging from 0 to 6 groups of diagnoses). The mean (SD) baseline CDR sum of boxes was 13.1 (4.0). Of the 932 participants at baseline, 160 (17.2%) participants were available at the fourth follow-up (see Figure 1). The individual development of P-ADL throughout the follow-up period and the mean P-ADL at four time points (see Table 2) indicated worsening in P-ADL functioning, which was confirmed by the second-order growth model. The positive linear time component (95% CI) of 0.14 (0.12; 0.16) shows that the P-ADL functioning declined over time, and negative second-order time component of -0.001 (-0.001: -0.0005) shows that the mean rate of decline decreased with time.

**Table 1:** Characteristics of study sample at baseline (N=932)

***Demographic***		**Total**	
Previous length of stay (days)	Mean *(SD)*	928.9	(910.1)
Women	N *(%)*	686	(73.6)
Age (year)	Mean *(SD)*	84.5	(7.5)
Single as marital status	N *(%)*	714	(79.4)
Ten years of school or less	N *(%)*	673	(74.1)
*Information on somatic health*			
Co-morbidity index^1^	Mean *(SD)*	1.8	(1.3)
Severely impaired hearing	N *(%)*	126	(13.6)
Severely impaired vision	N *(%)*	139	(14.9)
*Cognitive functioning*			
CDR sum of boxes	Mean *(SD)*	13.1	(4.0)
*Behavioral and psychological sub-syndromes of dementia (12-item NPI)*			
Agitation	Mean *(SD)*	6.5	(8.2)
Psychosis	Mean *(SD)*	3.2	(5.4)
Affective	Mean *(SD)*	3.7	(5.4)
Apathy	Mean *(SD)*	2.4	(3.8)
*Psychotropic medication*^*2*^			
Antipsychotics	N *(%)*	241	(25.9)
Antidepressants	N *(%)*	364	(39.1)
Anxiolytics	N *(%)*	221	(23.7)
Sedatives	N *(%)*	245	(26.3)
Antidementia	N *(%)*	126	(13.5)

CDR= Clinical Dementia Rating Scale.

^1^The co-morbidity index was calculated by summing up the occurrence of diagnoses (yes/no) in nine groups of diagnoses.

^2^The cumulative proportion of psychotropic medication is higher than 100 due to combinational use of psychotropic medication.

**Table 2:** P-ADL score at four time points

**Time point**	**Min, Max**	**Mean (SD)**
T1	6, 29	18.8 (5.3)
T2	7, 30	19.6 (5.2)
T3	7, 30	20.8 (4.8)
T4	9, 29	21.6 (4.6)

### The importance of degree of dementia for P-ADL functioning

The importance of degree of dementia for P-ADL functioning over time was studied in two adjusted models. In the first adjusted model (Table 3), the association between degree of dementia at baseline and P-ADL functioning over time was assessed. There was a significant second-order time trend in P-ADL functioning, and more severe dementia at baseline was associated with declined P-ADL functioning over time. The association between degree of dementia and P-ADL functioning was strongest at baseline, the development in time flattened and the association between the baseline degree of dementia and the decline in P-ADL functioning decreased with time.

**Table 3:** Model 1: Effects of cognitive impairment (CDR sum of boxes) measured at baseline on P-ADL level over time estimated by linear mixed model with random effects for intercepts and time

**In dependent variables**	**Unadjusted regression coefficients**		**Adjusted regression coefficients**	
	**Coeff. (95% CI)**	**p-value**	**Coeff. (95% CI)**	**p-value**
***Effect of main variables***^1^				
Time	0.19 (0.15; 0.24)	**<0.001**	0.20 (0.16; 0.25)	**<0.001**
Time* time	-0.001 (-0.001; -0.0005)	**<0.001**	-0.001 (-0.002; -0.001)	**<0.001**
CDR sum of boxes	0.72 (0.65; 0.79)	**<0.001**	0.61 (0.53; 0.69)	**<0.001**
Time* CDR sum of boxes	-0.004 (-0.007; -0.001)	**0.007**	-0.004 (-0.007; -0.001)	**0.013**
***Effect of additional variables at baseline***^2^				
Length of stay	0.002 (0.001; 0.002)	**<0.001**	0.001 (0.001; 0.001)	**<0.001**
*Socio –demographic information*				
Age (years)	-0.02 (-0.06; 0.02)	0.312	-0.05 (-0.09; -0.01)	**0.007**
Women	-0.33 (-1.03; 0.38)	0.362	-0.62 (-1.25; 0.01)	0.054
Single	-0.80 (-1.58; -0.03)	**0.042**	-0.72 (-1.41; -0.03)	**0.041**
Ten years of education or less	0.46 (-0.25; 1.17)	0.206	0.48 (-0.12; 1.07)	0.117
*Somatic health difficulties*				
Co-morbidity index	0.21 (-0.04; 0.46)	0.100	0.32 (0.11; 0.53)	**0.003**
No severe vision impairment	1.65 (0.77; 2.53)	**<0.001**	0.93 (0.19; 1.67)	**0.014**
No severe hearing impairment	-0.79 (-1.70; 0.13)	0.091	-0.12 (-0.90; 0.66)	0.765
*Degree of neuropsychiatric problems*				
Agitation sub-syndrome	0.08 (0.04; 0.12)	**<0.001**	0.01 (-0.0; 0.04)	0.546
Psychosis sub- syndrome	0.04 (-0.02; 0.10)	0.150		
Affective sub-syndrome	0.02 (-0.04; 0.07)	0.602		
Apathy	0.35 (0.27; 0.43)	**<0.001**	0.10 (0.03; 0.18)	**0.007**
*Use of psychotropic medication*				
Antipsychotics	0.72 (0.01; 1.42)	**0.046**	-0.08 (-0.69; 0.53)	0.802
Antidepressants	-0.56 (-1.19; 0.07)	0.081	-0.33 (-0.86; 0.20)	0.225
Anxiolytics	-0.28 (-1.01; 0.44)	0.443	-0.70 (-1.31; -0.10)	**0.024**
Sedatives	-0.65 (-1.36; 0.05)	0.070	-0.12 (-0.70; 0.47)	0.701
Cognitive enhancers	-3.23 (-4.09; -2.36)	**<0.001**	-1.81 (-2.58; -1.05)	**<0.001**

^1^The coefficients of the main effect variables (time, squared time, CDR sum of boxes, and interaction between time and CDR sum of boxes) unadjusted for other independent variables.

^2^The coefficients (95%CI) for single independent variables from the model containing only linear and second-order time components.

* = Multiplied with Bold data indicate p-value <0.05.

Table 4 presents the results from the second model finding degree of dementia at each time point being associated with poorer P-ADL functioning at the same time points. As in the first model, there was a decline in P-ADL functioning throughout the follow-up period. Furthermore, as in the first model, the association between degree of dementia and P-ADL functioning was strongest at baseline, the development in time flattened and the association between baseline degree of dementia on decline in P-ADL functioning became weaker with time.

**Table 4:** Model 2: Effects of cognitive impairment (CDR sum of boxes) measured at four time points on P-ADL level at the same time points estimated by linear mixed model with random effects for intercepts and time

	**Unadjusted regression coefficients**		**Adjusted regression coefficients**	
	**Coeff. (95% CI)**	**p-value**	**Coeff. (95% CI)**	**p-value**
***Effect of main variables***^1^				
Time	0.06 (0.02; 0.10)	**0.002**	0.18 (0.14; 0.22)	**<0.001**
Time* time	0.001 (-0.001; -0.001)	**<0.001**	-0.001 (-0.001; -0.0004)	**<0.001**
CDR sum of boxes (at 4 time points)	0.48 (0.43; 0.54)	**<0.001**	0.60 (0.52; 0.67)	**<0.001**
Time* CDR sum of boxes	0.003 (0.001; 0.006)	**0.007**	-0.003 (-0.006; -0.0006)	**0.019**
***Effect of additional variables at baseline***^2^				
Length of stay	0.002 (0.001; 0.002)	**<0.001**	0.001 (0.001; 0.0011)	**<0.001**
*Socio –demographic information*				
Age (years)	-0.02 (-0.06; 0.02)	0.312	-0.04 (-0.08; -0.004)	**0.030**
Women	-0.33 (-1.03; 0.38)	0.362	-0.51 (-1.13; 0.11)	0.108
Single	-0.80 (-1.58; -0.03)	**0.042**	-0.78 (-1.45; -0.11)	**0.023**
Ten years of education or less	0.46 (-0.25; 1.17)	0.206	0.45 (-0.14; 1.03)	0.136
*Somatic health difficulties*				
Co-morbidity index	0.21 (-0.04; 0.46)	0.100	0.35 (0.15; 0.56)	**0.001**
No severe vision impairment	1.65 (0.77; 2.53)	**<0.001**	0.88 (0.14; 1.61)	**0.019**
No severe hearing impairment	-0.79 (-1.70; 0.13)	0.091	-0.16 (-0.92; 0.61)	0.689
***Effect of additional variables assessed at 4 time-points***^2^				
*Degree of neuropsychiatric problems*				
Agitation sub-syndrome	0.04 (0.02; 0.06)	**<0.001**	0.03 (0.003; 0.05)	**0.026**
Psychosis sub- syndrome	0.02 (-0.01; 0.06)	0.173		
Affective sub-syndrome	0.01 (-0.03; 0.04)	0.614	-0.03 (-0.06; 0.01)	0.166
Apathy	0.20 (0.15; 0.24)	**<0.001**	0.12 (0.08; 0.17)	**<0.001**
*Use of psychotropic medication*				
Antipsychotics	0.77 (0.31; 1.23)	**0.001**	0.41 (-0.03; 0.86)	0.067
Antidepressants	-0.63 (-1.05; -0.20)	**0.004**	-0.40 (-0.80; 0.003)	0.052
Anxiolytics	-0.67 (-1.15; -0.20)	**0.005**	-0.77 (-1.22; -0.32)	**0.001**
Sedatives	-0.29 (-0.75; 0.17)	0.213		
Cognitive enhancers	-2.31 (-2.94; -1.67)	**<0.001**	-1.72 (-2.33; -1.11)	**<0.001**

^1^The coefficients of the main effect variables (time, squared time, CDR sum of boxes, and interaction between time and CDR sum of boxes) unadjusted for other independent variables.

^2^The coefficients (95%CI) for single independent variables from the model containing only linear and second-order time components.

* = Multiplied with Bold data indicate p-value <0.05.

In addition, longer length of stay, being married, having a higher comorbidity index score and having severely impaired vision at baseline were associated with worse P-ADL functioning through the observation period. Among the independent variables with assessments at all four time points included in the analysis, a higher agitation sub-syndrome score and apathy score, as well as not using anxiolytics and antidementia medication were associated with lower P-ADL functioning at the same time points.

**Figure 2:**
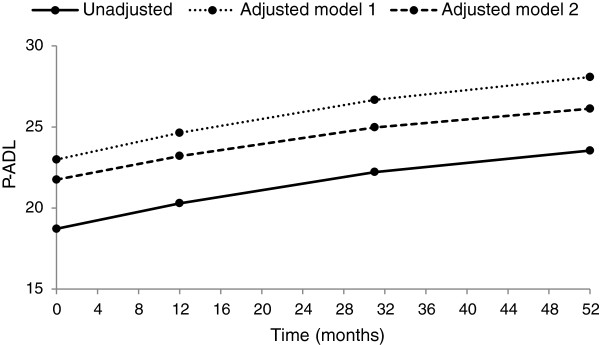
**Development of P-ADL score in time, unadjusted, adjusted in Model 1 and adjusted in Model 2.** P-ADL values demonstrate a significant second-order time trend indicating that the P-ADL functioning declines at the start of the follow-up period and flattens out later. Adjustment of time trend for clinical and demographic characteristics resulted only in minor changes.

Figure 2 illustrates the unadjusted P-ADL development over the follow-up period as well as P-ADL development adjusted in two different ways (two models). Independently of the adjustments made, there is an upward trend in the P-ADL values over time, i.e. the P-ADL functioning declines, but the rate of decline flattened during follow-up. The degree of decline in P-ADL functioning during follow-up was moderated using the adjusted models.

## Discussion

This follow-up study of 932 nursing home residents with dementia found that degree of dementia at baseline and the course of dementia as measured by the CDR during follow-up was significantly associated with lower P-ADL functioning. The rate of decline in P-ADL functioning explained by the degree of dementia decreased during follow-up. Independently of the adjustments made, there is a decline in P-ADL function over time and the rate of decline due to time of follow-up decreased during follow-up.

Other authors have pointed to a relationship between more severe dementia and worse P-ADL both among older hospitalized patients [11], community dwelling older persons [9,10] and in nursing home residents [23,26,28]. However, few have studied the P-ADL development and slope of development of P-ADL by time and degree of dementia in nursing home residents with dementia using several years of follow-up. Our results are supported by a six month follow-up study among residents with dementia. In this study, the mean P-ADL reduction increased in residents with severe dementia [26]. Another nursing home study in the USA, which followed residents with and without dementia for one year, explored if the degree of cognitive impairment at baseline affected the rate of P-ADL decline during follow-up [31]. This study with a follow-up period of 12 months did not find that cognitive impairment at baseline affected the rate of decline in P-ADL functioning over time, but a low P-ADL functioning at baseline was explained by baseline cognition [31]. However, a recent Swedish community study of older persons diagnosed with minimal cognitive impairment or Alzheimer disease reported that the rate of decline in P-ADL functioning due to degree of cognitive impairment decreased during the follow-up period of eight years, i.e. the slope of decline in P-ADL functioning leveled off [55]. Our results as well as the results of the Swedish study indicate the importance of “tailoring” the care for persons with dementia as the dementia disorder progress and the P-ADL decrease. The tailoring of care according to the patients increasing needs will probably increase the quality of life for the persons with dementia [56].

Interestingly, we found that worse P-ADL functioning at each time point was associated with a higher level of neuropsychiatric symptoms at the same time points, i.e. higher agitation sub-syndrome or apathy symptoms. Our findings are in line with previous research. Previously, it has been reported that a higher baseline sum score of neuropsychiatric symptoms is associated with a higher tendency for falls the coming year [33] and apathy symptoms have been reported to be associated with more motor and process skills difficulties [34]. The present study found an association between higher P-ADL functioning and the use of anti-dementia medication. This is in line with the findings of many randomized controlled trials that have shown an effect of treatment with antidementia medication on P-ADL [20,36,37]. However, residents using anti-dementia medication may have higher P-ADL functioning of other reasons which we did not adjust for. Why the use of anxiolytics should have a positive effect on P-ADL functioning is not easy to explain, but it could be that by reducing anxiety in patients with dementia, especially in those with agitation, their concentration and activity can improve.

In contrast to prospective studies which have not studied the importance of physical health in nursing home residents for the association between degree of dementia and P-ADL functioning or have excluded residents with co-morbid physical disorders [26,29-32], the present study adjusted for co-morbid physical disorders. In line with previous known risk factors for the decline of P-ADL over time, we found that higher co-morbidity and severe vision impairment at baseline were associated with lower P-ADL functioning at each time point [10]. This knowledge should also be used when tailoring the care for persons with dementia according to their needs.

Even if the study has a number of advantages, such as using a well-known, internationally accepted P-ADL measure, having a high number of baseline residents and adjustment for a high number of variables of potential importance to the outcome, such as health and demographic variables, there are some limitations that need to be addressed. Firstly, associations found in our study should be interpreted with caution since our design does not allow for inferences about causality.

Secondly, assessments of P-ADL functioning of older persons in a longitudinal study with several follow-ups over a period of years are statistically challenging. Repeatedly measured P-ADL functioning for the same individuals implies dependency in data. Having a large number of drop-outs, due to death leads to a varying number of observations per individual and generates unbalanced longitudinal data. Furthermore, a number of the independent variables in the adjusted models are time-varying. However, the linear mixed model used is particularly flexible with respect to these issues. The model handles any degree of imbalance in data by including all available observations. It also accounts for the correlations among repeated measurements in a relatively parsimonious way.

Thirdly, even if information about use of each category of psychotropic medications were available, we did not have the dosages of the psychotropic medications. Moreover, use of a well-known comorbidity index, f. ex. Charlson comorbidity index [57,58] rather than the comorbidity index we applied would have been more informative. Lastly, it may be questioned if the Minimal Mental State Examination [59,60] widely used and accepted could have been a better choice for the assessment dementia than CDR which we applied. The MMSE has the advantage of providing a direct measure of cognition. However, in a sample as ours many will have scores in the lower range, and previous research has questioned the ability of the MMSE to differentiate between persons with severe dementia [61]. As a consequence of these limitations, we cannot rule out that there are confounders which we have not adjusted adequately for.

## Conclusion

In a large-scale longitudinal nursing home study among patients with dementia, using four assessments during a period of 52 months, we found that P-ADL functioning worsened over time. The worsening of P-ADL functioning was associated with a higher baseline degree of dementia and with an increasing degree of dementia during follow-up. The importance of degree of dementia and follow-up time for degree of decline in P-ADL functioning decreased during follow-up.

Clinicians should pay attention to the associates of P-ADL functioning identified in our study in order to help the nursing home residents with dementia maintain their level of functioning as long as possible.

## Competing interests

The authors declare that they have no competing interests.

## Authors’ contributions

GS and KE designed the study, and GS was responsible for the data collection and quality as e study results. JSB analyzed the majority of the data for this manuscript. ASH has analyzed sections of the data and drafted the manuscript. All authors participated in the interpretation of the study results and in editing the manuscript, and have read and approved the final manuscript.

## Pre-publication history

The pre-publication history for this paper can be accessed here:

http://www.biomedcentral.com/1471-2318/14/45/prepub
